# Intention of smartphone application usage in diabetes self-management and its associated factors among patients with diabetes: A cross-sectional study

**DOI:** 10.51866/oa.729

**Published:** 2025-02-21

**Authors:** Geok Seim Lim, Ai Theng Cheong, Ping Yein Lee, Shariff Mohamad Siti Maisarah

**Affiliations:** 1 MBBS, MMed (Family Medicine), PhD, Department of Family Medicine, Faculty of Medicine and Health Sciences, Universiti Putra Malaysia Serdang, Selangor, Malaysia. Email: cheaitheng@upm.edu.my; 2 MD, MMed (Family Medicine), Kementerian Kesihatan Malaysia, Klinik Kesihatan Mahmoodiah, JKR 6274, Jalan Mahmoodiah, Johor Bahru, Johor, Malaysia.; 3 MBBS, MMed (Family Medicine), UMeHealth Unit, Faculty of Medicine, Universiti Malaya, Malaysia.; 4 MD, Department of Family Medicine, Faculty of Medicine and Health, Sciences, Universiti Putra Malaysia Serdang, Selangor, Malalysia.

**Keywords:** Diabetes mellitus, Selfmanagement, Primary health care, Mobile health

## Abstract

**Introduction:**

Many Malaysians with diabetes lack sufficient knowledge about diabetes selfmanagement. With the widespread adoption of smartphones, mobile health (mHealth) solutions could help empower patients with diabetes to self-manage their condition effectively. This study aimed to determine the intention of patients with diabetes to use diabetes self-management applications (DSMAs) and its associated factors in a primary care setting.

**Methods:**

A cross-sectional study was conducted at a government health clinic in the Federal Territory of Kuala Lumpur from 1 July to 30 September 2019. We recruited 723 patients with diabetes using systematic random sampling. A validated self-administered questionnaire was used to evaluate patients’ intention to use DSMAs and its associated factors were determined via multiple logistic regression.

**Results:**

Among 719/723 patients with diabetes included in the analysis, 49.9% intended to use DSMAs. Those who had a household income of >RM 6000 (adjusted odds ratio [AOR] = 10.652, 95% confidence interval [CI] = 1.709-66.398, P<0.011), agreed (AOR=8.959, 95% CI=3.327- 24.128, P<0.001) or neutrally agreed (A0R=3.403, 95% CI= 1.188-9.749, P=0.023) with the perceived usefulness of DSMAs, did not have resistance to change (A0R=2.458, 95% CI= 1.2934.672, P=0.006) and had a facilitating condition (A0R=9.454, 95% CI=2.718-32.881, P<0.001) had higher odds of intending to use DSMAs than their counterparts.

**Conclusion:**

Nearly half of patients with diabetes intend to use DSMAs, indicating the potential of DSMAs as alternative tools for assisting in diabetes self-management. Education focusing on the usefulness of DSMAs and exploring facilitating conditions with patients can help increase the intention of patients to use DSMAs.

## Introduction

Diabetes mellitus is a non-communicable disease that is associated with multiple macrovascular and microvascular complications,^1^ which can lead to preventable morbidity and premature mortality.^2^ According to the International Diabetes Federation (IDF), Malaysia had 22,130,900 people with diabetes in 2021, among whom 20% were adults. The IDF estimates that by 2045, the number of people with diabetes in the Western Pacific region will increase to 260 million.^3^ Hence, diabetes remains one of the major concerns in Malaysia’s public health system currently and in the future.

Apart from the rising diabetes prevalence in Malaysia, glycaemic control among patients with diabetes in public hospitals,^4^ private primary healthcare settings^5^ and public primary healthcare settings^6^ remains unsatisfactory. In terms of diabetes care delivery in primary care settings, only 23.3% of people with type 2 diabetes achieve a haemoglobin A1c (HbA1c) level below 6.5%.^7^

To achieve good glycaemic control, both healthcare professionals and patients play an important role. Patients who have a better understanding of their disease will have better glycaemic control, as their medication adherence is better.^8^ Diabetes self-management involves patients actively monitoring their blood sugar levels, adhering to medication, maintaining a healthy diet, exercising and managing stress. It encompasses being proactive in diabetes care to prevent complications and improve quality of life.^9^ Thus, diabetes self-management education is essential for the management of diabetes to optimise glycaemic control and prevent disease complications.

The Malaysia Clinical Practice Guideline on the Management of Type 2 Diabetes Mellitus^10^ has recommended that education for diabetes selfmanagement should be advocated for all patients with type 2 diabetes mellitus regardless of their treatment mode. Diabetes educators could guide and support all patients with diabetes in selfmanagement, which subsequently helps in better glycaemic control.^11^

However, many Malaysians with diabetes have insufficient knowledge regarding diabetes self- management,^12^ poor dietary practices,^12,13^ sedentary lifestyle,^13^ poor adherence to medications^12,14^ and poor sugar monitoring.^12,15^ In addition, there is a significant shortage of diabetes educators in Malaysia.^13^ This yields a lack of supervision, guidance and support for diabetes self-management.^13^

Thus, new approaches are needed to improve patient engagement in diabetes self-management to optimise treatment and reduce the risk of complications. The latest advancements and the rapid adoption of mobile phone technologies have been applied to medical technology.^16^ This has further enhanced the medical field in combating chronic diseases, and the application is referred to by some groups as mobile health (mHealth).^16^ The World Health Organization defined mHealth as medical and public health practice supported by mobile devices, such as mobile phones, patient monitoring devices, personal digital assistants and other wireless devices.^16^ mHealth allows patients to be connected to services that include health information and demand, health record management and remote, real-time monitoring of chronic conditions such as diabetes, asthma and hypertension.^17^

The intention to use diabetes self-management applications (DSMAs) is crucial, as it serves as a precursor to actual usage, which directly impacts patients’ outcomes. Patients who use DSMAs are more likely to engage in self-management practices, leading to better glycaemic control and overall health.^18,19^ In our study, we employed the health belief model and theory of planned behaviour as the theoretical framework, which helps explain how beliefs about health risks and benefits influence behavioural intentions.^20,21^ Early evidence from several studies supports the effectiveness of health-related text messages and mHealth applications in improving diabetes self-management behaviours. The meta-analysis conducted by Hou et al. across 14 studies with 1360 participants showed a significant reduction of the A1c level among patients with diabetes using mobile phone applications compared to controls.^19^

Research on the factors influencing the intention to use mHealth has indicated that sex, age, educational level and clinical characteristics play varying roles depending on the study population and location.^22-26^ The studies conducted by Hussein et al. in Sarawak and Rai et al. in the US found sex as an insignificant factor in determining mHealth usage, although other studies have suggested that female patients show more interest in self-managing diabetes via applications.^22,25^ Age presents mixed findings: Hussein et al. reported no significant impact in Sarawak, while the studies performed by Wang et al. in Korea, Japan and the US showed that younger individuals were more likely to use mHealth for diabetes management.^22-24,26^

Educational level similarly shows contrasting findings: While Hussein et al. and Shibuta et al. found no significant correlation, Wang et al. observed that a higher educational level was linked to greater mHealth usage.^22,23,26^ In terms of clinical characteristics, Shibuta et al. discovered that in Japan, patients with hypertension and without nephropathy were more willing to use mHealth tools, while those with dyslipidaemia, cerebrovascular diseases and cardiovascular diseases showed less interest.^23^ No significant association was noted between diabetes control and mHealth usage in both the Japanese and Sarawak studies.^22,23^

The use of smartphones is significantly associated with the intention to adopt mHealth applications for diabetes management. According to Shibuta et al., Humble et al. and Wang et al., smartphone users are more likely to adopt such applications.^23,24,26^ Perception factors, including ease of use, usefulness, privacy and security risks, financial concerns and technology anxiety (TA), play a critical role in this adoption. For instance, Byomire and Maiga and El-Wajeeh et al. found that perceived ease of use (PEOU) and perceived usefulness (PU) strongly influenced adoption.^27,28^ However, the local study by Maniam et al. reported that ease of use and usefulness were not significant predictors among patients with diabetes in Malaysia.^29^ Privacy, financial risk and TA are other important factors influencing the adoption of DSMAs.^24,29,30^ Facilitating conditions (FCs) and resistance to change (RC) also impact users’ intentions, with studies highlighting that access to resources and knowledge significantly encourage adoption.^29,31,32^

With the growing population of patients with diabetes and the shortage of healthcare providers to guide patient self-management, the use of mHealth may be a viable solution to improve self-management among patients with diabetes in Malaysia. Awareness of DSMAs is an important factor for patients considering using them for their diabetes management. However, such awareness may not necessarily translate into usage, and there are limited studies on the intention to use mHealth for diabetes self-management among patients in Malaysia. Therefore, our study aimed to determine the intention of patients with diabetes to use DSMAs and its associated factors in a primary healthcare setting.

## Methods

### Study design

A cross-sectional study was conducted at a government health clinic in the Federal Territory of Kuala Lumpur. This clinic was chosen owing to the large number of patients with active diabetes, with approximately 5455 patients with diabetes attending the clinic annually. Data were collected from 1 July to 30 September 2019.

### Study population

All patients attending the health clinic during the study period who were aged 18 years or older and had a confirmed diagnosis of diabetes mellitus documented in their case notes were included in the study. Patients who were unable to communicate their response to survey questions, those with severe visual impairment and those experiencing acute illness requiring emergency treatment during their clinic visit were excluded from the study.

### Sample size calculation

The sample size was calculated based on the study by Shibuta et al.^23^ For the intention to use DSMAs, the single-proportion formula was used, and the estimated sample size was 384 based on a 50% prevalence of patients with diabetes who were willing to use an information and communication technology (ICT) selfmanagement tool and a 95% confidence interval (CI). For the associated factors, the sample size was determined using a 95% CI and a 5% margin of error, applying the formula for estimating proportions between two populations^33^ based on the proportions of patients with diabetes and nephropathy willing (25.5%) and not willing to use ICT (36.4%). According to this calculation, the estimated sample size was 561, but a minimum total of 701 respondents were required after accounting for a 20% non-response rate.

### Data collection

Participants were recruited through systematic random sampling. The first potential respondent was selected using a lottery method (rolling dice), and the subsequent respondents were chosen at an interval of two using the sampling fraction formula. The sampling fraction (k) for the sample size was obtained by dividing the estimated number of patients attending the diabetes clinic of the health clinic during the study period (N=1365) by the total number of participants required in this study (N=701). The first respondent was the patient who received number 6 with the rolling dice. The subsequent respondents were the patients who received numbers starting from number 6 to 8, 10, 12 and so on. They were required to complete a set of self-administered questionnaires. Participants had no time limit for completing the questionnaire and could seek clarification from the researchers when they had any questions.

### Research instrument and scoring method

The questionnaire comprised four sections: 1) Section A focused on participants’ demographic information including age, sex, ethnicity, educational level and household income. 2) Section B explored the accessibility to technologies including current handphone or smartphone usage, frequency of handphone usage, experience of mobile application usage and awareness of DSMAs. 3) Section C investigated the perceptions towards the use of diabetes selfmanagement mobile applications and intention to use DSMAs. 4) Section D covered the clinical characteristics including body mass index (BMI), diabetes duration, diabetes control, diabetes medications, diabetes complications and comorbidities. The questions in section C were based on the validated questionnaire from the local study by Maniam et al.^29^ This questionnaire consisted of seven domains of perceptions towards DSMAs, which included PEOU, PU, perceived financial risk (PFR), perceived privacy and security risk (PR), TA, RC and FC, with excellent internal consistency (Cronbach’s alpha coefficient=0.953-0.995), and a domain for the intention to use DSMAs, with a Cronbach’s alpha coefficient of 0.993.^29^ All items in the questionnaire were measured using a Likert scale consisting of five response choices ranging from ‘strongly disagree’ (score of 1) to ‘strongly agree’ (score of 5). A higher mean score within a domain indicated greater agreement with the related domain.

The intention to use DSMAs was defined as motivation or willingness to engage with and utilise mobile applications on smartphones for diabetes self-management regardless of current access to smartphones. The accessibility to technologies was described as whether participants were handphone or smartphone users and based on the frequency of usage and their awareness of DSMAs. The perception towards DSMAs referred to the PEOU, PU, PFR, PR, TA, RC and FC towards DSMAs.

A handphone was defined as any portable phone that can be used while holding it in the hand. It included both feature phones and smartphones. A feature phone referred to a basic mobile phone that provides essential functions such as calling, texting and using a few basic applications. It lacks the advanced capabilities of a smartphone, typically no touch screens, and runs on simpler operating systems. A smartphone was described as a more advanced mobile phone that combines the functionality of a phone with that of a computer. It has touch screens, internet access and cameras and runs on sophisticated operating systems such as iOS or Android. It also supports a wide range of applications and is designed for more than just calling and texting.

For BMI, participants’ height and weight were obtained from their case notes. If these were not documented, the body mass was divided by the square of the body height. BMI is universally expressed in units of kg/m^2^, with mass in kilograms and height in metres. Following the 2004 Malaysian Clinical Practice Guideline for the Management of Obesity, BMI was classified as follows: <18.5 kg/m^2^ (underweight), 18.522.9 kg/m^2^ (normal weight), 23.0-27.4 kg/m^2^ (overweight), 27.5-34.9 kg/m^2^ (obesity I), 35.0-39.9 kg/m^2^ (obesity II) and ≥40.0 kg/m^2^ (obesity III).^34^

### Pre-test study

The questionnaire was pre-tested on the target population prior to the actual period of data collection. Seventy participants were included in the pre-test. The purpose of this pre-test was to assess the recruitment process, evaluate the face validity of the questionnaire and identify any potential issues that might arise during data collection. During the pre-test, the researchers noticed that some older adult patients did not complete the questionnaire due to a lack of understanding of mobile applications or DSMAs. Hence, either the researchers or research assistants provided further explanation regarding DSMAs to participants as required. After 10 minutes of explanation, their understanding of the subject significantly improved, and they were able to complete the questionnaire. During the study data collection, additional explanation was provided by the researchers to older adult participants.

### Data analysis

Data were recorded and analysed using SPSS version 21. The dependent variable was the intention to use DSMAs, which was not normally distributed. It was categorised into two groups using the median score of 3.00 as the cut-off point, with a score of >3.00 indicating greater intention and a score of ≤3.00 indicating lesser intention.^35^

The independent variables were the sociodemographic and clinical characteristics (i.e. age, sex, race, educational level, household income, BMI, duration of diabetes, level of glycaemic control, medication, number of complications, diabetic retinopathy, diabetic neuropathy, diabetic nephropathy, macrovascular disease and type of comorbidities), accessibility to technologies (i.e. handphone user, type of current phone used, frequency of handphone usage, ever use of smartphone applications and awareness of DSMAs) and perception towards DSMAs (i.e. PEOU, PU, PFR, PR, TA, RC and FC). For perception towards DSMAs, the mean score was calculated and regrouped into the following three groups: disagree (score of 0.00-2.00), neutral (score of 2.01-3.00) and agree (score of 3.01-5.00).^29,35^

The continuous data were not normally distributed. Hence, they were converted to categorical data, and medians and interquartile range (IQRs) were used to report them. Frequencies and percentages were used to describe the categorical data. The chi-square test was utilised to determine the association of the intention to use DSMAs with the sociodemographic characteristics, clinical characteristics, accessibility to technologies and perception towards DSMAs. Multicollinearity of the independent variables was tested by examining the variance inflation factor (VIF), and the VIF value for the independent variables was all below 10 (range= 1.056-7.708); this indicated no collinearity between the independent variables.^36^ Univariate logistic regression analysis was performed, and the factors with a P-value of <0.25 in this analysis were included in the multivariate model.^37^ The results of both univariate and multivariate logistic regression analyses were presented as odds ratios (ORs) with 95% CIs.

## Results

A total of 723 participants responded to the questionnaire. However, four participants were excluded from the analysis, as there was missing information regarding the dependent variable. Thus, a total of 719 participants were included in this study. The median age of the participants was 59.33 (IQR=11.32) years. About half of the participants (52.9%) were older adults, and 81.5% (581/713) had a household income of <RM 3000. Most participants (520/716) were diagnosed with diabetes with a duration of ≥5 years. Approximately 65.3% (461/706) had an HbA1c level of >8%, and the majority had comorbidities (95.6%) and diabetes complications (67.2%). (Refer Table 1).

**Table 1:** Sociodemographic and clinical characteristics of the study participants.

Variable		n	%
**Sociodemographic characteristics**			
Age, year	<40	37	5.1
(n=719)	40-59	302	42.0
	60-75	320	44.5
	>75	60	8.4
Sex	Male	299	41.6
(n=719)	Female	420	58.4
Race	Malay	218	30.3
(n=719)	Chinese	329	45.8
	Indian	169	23.5
	Others	3	0.4
Educational level	No formal education	73	10.2
(n=717)	Primary	241	33.6
	Secondary	342	47.7
	Pre-university	23	3.2
	Tertiary	38	5.3
Household income, RM	<3000	581	81.5
(n=713)	3001-6000	108	15.1
	6001-9000	15	2.1
	9001-12,000	4	0.6
	>12,000	5	0.7
**Clinical characteristics**			
BMI, kg/m^2^ (n=699)			
Underweight	<18.5	5	0.7
Normal weight	18.5-22.9	85	12.2
Overweight	23.0-27.4	261	37.3
Obesity I	27.5-34.9	269	38.5
Obesity II	35.0-39.9	53	7.6
Obesity III	>40.0	26	3.7
Diabetes duration, year	<5	196	27.4
(n=716)	5-10	217	30.3
	>10	303	42.3
HbA1c level, %	≤6.5	65	9.2
(n=706)	6.6-7.0	54	7.6
	7.1-7.5	47	6.7
	7.6-8.0	79	11.2
	8.1-10.0	265	37.5
	10.1-12.0	139	19.7
	>12.0	57	8.1
Diabetes medications	OHA only	271	37.8
(n=717)	Insulin only	49	6.8
	OHA and insulin	395	55.1
	No medication	2	0.3
Number of complications	1	257	35.8
(n=719)	2	169	23.5
	≥3	57	7.9
	0	236	32.8
Diabetes complications^c^	Retinopathy	296	41.2
(n=719)	Nephropathy	141	19.6
	Neuropathy	227	31.6
	Macrovascular	108	15.0
Comorbidities	HPT only	66	9.2
(n=719)	HPL only	107	14.9
	HPT and HPL	514	71.5
	None	32	4.4

c Each patient with diabetes might have one or more diabetes complications. OHA: oral hypoglycaemic agent, HPT: hypertension, HPL: dyslipidaemia

### Intention to use DSMAs

Among the participants, 49.9% (359/719) had greater intention to use DSMAs, while 50.1% (360/719) had lesser intention to use DSMAs.

### Accessibility to technologies

The majority of the participants (86.9%, 625/719) were handphone users, while 64.5% (464/719) were smartphone users. Most participants (65.8%, 473/719) were using their handphones two times or more in a day. Although more than half of the participants (56.6%, 407/719) were using smartphone applications, only 3.3% (24/719) were aware of any DSMA.

### Perception towards DSMAs

Table 2 displays the participants’ perceptions towards DSMAs including the PEOU, PU, PFR, PR, TA, RC and FC. About three-fifths of the participants had PU (55.6%) and FC (58.8%) but no PR (55.0%), TA (60.1%) and RC (54.1%). However, about one-third indicated no PEOU of DSMAs (27.7%) and had financial concerns (37.0%).

**Table 2:** Perception towards diabetes self-management applications.

Variable	Frequency (N=719)	%	Median	IQR
PEOU			3.00	2.00
Disagree	200	27.7		
Neutral	175	24.4		
Agree	344	47.9		
PU			4.00	1.00
Disagree	160	22.2		
Neutral	159	22.2		
Agree	400	55.6		
PFR			3.00	2.00
Disagree	298	41.5		
Neutral	150	20.9		
Agree	267	37.0		
Missing	4	0.6		
PR			2.00	1.00
Disagree	396	55.0		
Neutral	178	24.8		
Agree	141	19.6		
Missing	4	0.6		
TA			2.00	1.00
Disagree	432	60.1		
Neutral	136	18.9		
Agree	150	20.9		
Missing	1	0.1		
RC			2.00	2.00
Disagree	389	54.1		
Neutral	93	12.9		
Agree	237	33.0		
FC			3.20	1.00
Disagree	129	18.0		
Neutral	164	22.8		
Agree	423	58.8		
Missing	3	0.4		

IQR: interquartile range, PEOU: perceived ease of use, PU: perceived usefulness, PFR: perceived financial risk, PR: perceived privacy and security risk, TA: technology anxiety, RC: resistance to change, FC: facilitating condition

### Association between the intention to use DSMAs and the sociodemographic characteristics, clinical characteristics, accessibility to technologies and perception towards DSMAs

All sociodemographic characteristics except for sex were significantly associated with the intention to use DSMAs (Table 3). Among the clinical characteristics, nephropathy, neuropathy and comorbidities were significantly associated with the intention to use DSMAs (Table 3). The accessibility to technologies (Table 3) and perception towards DSMAs were also significantly associated with the intention to use DSMAs (Table 4).

**Table 3:** Association between the sociodemographic characteristics, clinical characteristics, accessibility to technologies and intention to use DSMAs.

Variable	Intention to use DSMAs				Chi-square	P-value
	Lesser intention (n=360)		Greater intention (n=359)			
	n	%	n	%
**Sociodemographic characteristics**						
Age, year (n=719)					57.795^^^	<0.001^*^
<40	11	29.7	26	70.3		
40-59	110	36.4	192	63.6		
60-74	194	60.6	126	39.4		
≥75	45	75.0	15	25.0		
Sex (n=719)					0.002^^^	0.965
Male	150	50.2	149	49.8		
Female	210	50.0	210	50.0		
Race (n=719)					68.907^^^	<0.001^*^
Malay	65	29.8	153	70.2		
Chinese	216	65.7	113	34.3		
Indian and others	79	45.9	93	54.1		
Educational level (n=717)					109.686^^^	<0.001^*^
No formal education	58	79.5	15	20.5		
Primary	166	68.9	75	31.1		
Secondary	123	36.0	219	64.0		
Pre-university	6	26.1	17	73.9		
Tertiary	6	15.8	32	84.2		
Household income, RM (n=713)					31.985^^^	<0.001^*^
<3000	321	55.2	260	44.8		
3001-6000	31	28.7	77	71.3		
>6000	6	25.0	18	75.0		
**Clinical characteristics**						
BMI, kg/m^2^ (n=699)					3.3000^^^	0.192
Underweight and normal weight	51	56.7	39	43.3		
Overweight	120	46.0	141	54.0		
Obese	176	50.6	172	49.4		
Duration of diabetes, year (n=716)					3.702^^^	0.157
<5	87	44.4	109	55.6		
5-10	110	50.7	107	49.3		
>10	161	53.1	142	46.9		
HbAlc level, % (n=706)					6.786^^^	0.148
≤6.5	39	60.0	26	40.0		
6.6-7.0	20	37.0	34	63.0		
7.1-7.5	25	53.2	22	46.8		
7.6-8.0	37	46.8	42	53.2		
>8.0	233	50.5	228	49.5		
Medication (n=717)					4.535^^^	0.104
OHA only and no medication	150	54.9	123	45.1		
Insulin only	25	51.0	24	49.0		
OHA and insulin	184	46.6	211	53.4		
Number of complications (n=719)					0.606^^^	0.895
1	128	49.8	129	50.2		
2	82	48.5	87	51.5		
≥3	31	54.4	26	45.6		
0	119	50.4	117	49.6		
Diabetes complications (n=719)						
Retinopathy	147	49.7	149	50.3	0.033^^^	0.855
Nephropathy	88	62.4	53	37.6	10.687^^^	0.001^*^
Neuropathy	96	42.3	131	57.7	8.029^^^	0.005^*^
Macrovascular	58	53.7	50	46.3	0.671^^^	0.413
Comorbidities (n=719)					13.121^^^	0.004^*^
HPT only	28	42.4	38	57.6		
HPL only	41	38.3	66	61.7		
HPT and HPL	279	54.3	235	45.7		
None	12	37.5	20	62.5		
**Accessibility to technologies**						
Handphone user (n=719)					43.866^^^	<0.001^*^
Yes	283	45.3	342	54.7		
No	77	81.9	17	18.1		
Type of handphone used (n=719)					106.030^^^	<0.001^*^
Smartphone	167	36.0	297	64.0		
Feature phone	116	72.0	45	28.0		
No handphone	77	81.9	17	18.1		
Frequency (n=714)					106.041^^^	<0.001^*^
≥2/day	178	37.6	295	62.4		
1/day	61	59.2	42	40.8		
<1/day	47	92.2	4	7.8		
0	72	82.8	15	17.2		
Prior use of applications (n=719)					100.391^^^	<0.001^*^
Yes	139	34.2	267	65.8		
No	144	65.8	75	34.2		
No handphone	77	82.8	17	18.1		
Awareness of DSMAs (n=718)					47.000^^^	<0.001^*^
Yes, aware	8	33.3	16	66.7		
Not aware	275	45.8	326	54.2		
No handphone	77	82.8	16	17.2		

^ Chi-square test

* Statistically significant

DSMA: diabetes self-management application, OHA: oral hypoglycaemic agent, HPT: hypertension, HPL: dyslipidaemia

**Table 4:** Association between the perception towards DSMAs and intention to use DSMAs.

Variable	Intention to use DSMAs				Chi-square	P-value
	Lesser intention		Greater intention			
	n	%	n	%
PEOU (n=719)					199.396^^^	<0.001^*^
Disagree	173	86.5	27	13.5		
Neutral	102	58.3	73	41.7		
Agree	85	24.7	259	75.3		
PU (n=719)					219.406^^^	<0.001^*^
Disagree	151	94.4	9	5.6		
Neutral	100	62.9	59	37.1		
Agree	109	27.3	291	72.8		
PFR (n=715)					29.356^^^	<0.001^*^
Disagree	114	38.3	184	61.7		
Neutral	90	60.0	60	40.0		
Agree	155	58.1	112	41.9		
PR (n=715)					41.419^^^	<0.001^*^
Disagree	159	40.2	237	59.8		
Neutral	122	68.5	56	31.5		
Agree	78	55.3	63	44.7		
TA (n=718)					62.676^^^	<0.001^*^
Disagree	165	38.2	267	61.8		
Neutral	89	65.4	47	34.6		
Agree	106	70.7	44	29.3		
RC (n=717)					125.255^^^	<0.001^*^
Disagree	123	31.6	266	68.4		
Neutral	53	58.2	38	41.8		
Agree	183	77.2	54	22.8		
FC (n=716)					215.035^^^	<0.001^*^
Disagree	122	94.6	7	5.4		
Neutral	118	72.0	46	28.0		
Agree	119	28.1	304	71.9		

^ Chi-square test

* Statistically significant

PEOU: perceived ease of use, PU: perceived usefulness, PFR: perceived financial risk, PR: perceived privacy and security risk, TA: technology anxiety, RC: resistance to change, FC: facilitating condition

### Determinants of the intention to use DSMAs

The variables with a P-value of <0.25 in the univariate logistic regression analysis were included in the multivariate logistic regression analysis. These variables were age, race, educational level, household income, diabetic nephropathy, diabetic neuropathy, comorbidities, handphone user, smartphone user, frequency of using handphones, experience of using smartphone applications, awareness of DSMAs, PEOU, PFR, PR, TA and RC.

The participants with a household income of >RM 6000 had 10.652 higher odds of intending to use DSMAs than those with a household income of <RM 3000 (adjusted odds ratio [A0R]=10.652, 95% CI= 1.709-66.398, P<0.011). The participants who agreed (A0R=8.959, 95% CI=3.327-24.128, P<0.001) or neutrally agreed (A0R=3.403, 95% CI=1.188–9.749, P=0.023) with the PU of DSMAs, did not have RC (A0R=2.458, 95% CI= 1.293–4.672, P=0.006) and had FCs (A0R=9.454, 95% CI=2.718–32.881, P<0.001) also had higher odds of intending to use DSMAs than their counterparts. (Refer Table 5)

**Table 5:** Multiple logistic regression analysis of the factors related to the intention to use DSMAs, adjusted for other variables (n=674).

Variable	B	SE	Wald	P-value	AOR	95% CI
**Household income, RM**						
<3000^#^					1	
3001-6000	0.367	0.334	1.209	0.271	1.443	0.750-2.777
>6000	2.366	0.934	6.421	0.011^*^	10.652	1.709-66.398
**PU**						
Disagree^#^					1	
Neutral	1.225	0.537	5.199	0.023^*^	3.403	1.188-9.749
Agree	2.193	0.505	18.823	<0.001^*^	8.959	3.327-24.128
**RC**						
Disagree	0.899	0.328	7.535	0.006^*^	2.458	1.293-4.672
Neutral	0.075	0.406	0.034	0.853	1.078	0.487-2.387
Agree^#^					1	
**FC**						
Disagree^#^					1	
Neutral	1.061	0.638	2.766	0.096	2.890	0.827-10.097
Agree	2.246	0.636	12.477	<0.001^*^	9.454	2.718-32.881

Controlled for age, race, educational level, monthly household income, medication, diabetic neuropathy, diabetic nephropathy, type of comorbidities, handphone user, type of current phone used, frequency of handphone usage, ever use of smartphone applications, awareness of DSMAs, PEOU, PFR, PR and TA Hosmer and Lemeshow test: P=0.816; Nagelkerke R-square=61.8%; the classification table shows 83.8% correct classification. No multicollinearity (variance inflation factor value ranging from 1.044 to 3.839) DSMA: diabetes self-management application, SE: standard error, CI: confidence interval, B: (3 coefficient, AOR: adjusted odds ratio, PEOU: perceived ease of use, PU: perceived usefulness, PFR: perceived financial risk, PR: perceived privacy and security risk, TA: technology anxiety, RC: resistance to change, FC: facilitating condition

# Reference group

* Statistically significant

## Discussion

In this study, we found that two-thirds (64.5%) of the patients with diabetes were smartphone users, but the usage and awareness of DSMAs were substantially low (3.3%). Nearly half (49.9%) expressed their intention to use DSMAs in the future. The determinants of having greater intention to use DSMAs were a household income of >RM 6000, the PU of DSMAs, the presence of FCs and the absence of RC.

The percentage of smartphone usage among the patients with diabetes in our study is lower than that among the general population in Malaysia, which was reported as 87.61% in 2020.^38^ This could be because more than half of our study participants were older adults, and the majority (81.5%) had a household income of <RM 3000.

Among the patients with diabetes in our study, only 3.3% were aware of DSMAs. A previous local study, which involved an online survey of 105 patients with diabetes, also reported a small proportion of patients (4.76%) having experienced using DSMAs.^29^ Similarly, a Canadian study reported a small proportion of patients with diabetes (7.1%) using a smartphone to help manage their diabetes. Our results indicate that more efforts are needed to create awareness and promote DSMAs to patients in public primary care clinics. A recent local qualitative study found a lack of awareness and recommendations regarding DSMAs from healthcare professionals.^39^ Emphasising the usefulness of DSMAs and addressing FCs can significantly increase patient intention to use these tools. General practitioners can play a crucial role by educating patients about the benefits of DSMAs, providing training and addressing any barriers to adoption.

Although the level of awareness of DSMAs was low in our study, half of the study participants expressed their intention to use DSMAs, indicating the possibility of using these tools to assist them in self-management. This is similar to literature from Japan and Iran, wherein 50% of patients with diabetes expressed their willingness and interest to use mHealth or ICT- based self-management tools for diabetes self- care.^23,40^

In our study, a higher household income was found to be a significant factor associated with greater intention to use DSMAs. Similarly, a study from China revealed that patients with a higher monthly income were more likely to use DSMAs.^41^ This could be because higher-income groups are more likely to afford smartphones to use DSMAs and have fewer concerns about the associated cost of using subscriptions to internet data.

Among the patients in this study, those who perceived the usefulness of DSMAs showed greater intention to use such applications. A systematic review of qualitative, mixed-method and cross-sectional studies suggested that patients would not use DSMAs if they do not perceive or are uncertain about the benefits of DSMAs.^42^ Thus, highlighting the usefulness of DSMAs to patients is important to increase their interest in adopting such tools in selfmanagement.

Having FCs (i.e. resources, knowledge and capabilities to seek help from others) was found to be a significant factor for greater intention to use DSMAs in our study. In their local study, Maniam et al. reported similar findings.^29^ Hence, patient education can potentially improve patients’ knowledge and capabilities in using DSMAs. For older adult patients, it is essential to explore their social support to help them navigate DSMAs, especially in the early stages of adopting these applications in selfmanagement.

In this study, we also found that RC significantly affected the patients’ intention to use DSMAs. Two previous studies also showed that RC was a main factor affecting the adoption of new technology including mHealth.^31,43^ If the user has high RC from their routine and usual practices, there will be a negative impact towards the intention to adopt DSMAs. Thus, having an FC can potentially help facilitate change.

## Strengths and limitations

To the best of our knowledge, this study is one of the few studies that examined the intention of patients with diabetes to use DSMAs and its associated factors. Additionally, the sample size is relatively larger than that of a local study.^29^

Several limitations must also be considered in this study. First, in view of resource constraints, this study was conducted at a public healthcare clinic, limiting the generalisation of the results to other settings with different patient profiles and resources. Second, a cross-sectional design was adopted; hence, causal relationships could not be determined. Third, social desirability bias may occur, potentially leading to an overestimation of the intention to use DSMAs. Finally, other factors that could influence patients’ intention to use DSMAs such as recommendations by healthcare providers or friends were not included in our study.

## Conclusion

Nearly half of patients with diabetes intend to use DSMAs, indicating the potential of DSMAs as alternative tools for assisting patients in diabetes self-management. Education focusing on the usefulness of DSMAs and exploring FCs with patients can help increase the intention of patients to use DSMAs. This study underscores the importance of personalised patient education and targeted interventions in promoting the adoption of digital health tools. By leveraging these insights, general practitioners can improve patient engagement, enhance self-management of diabetes and ultimately achieve better health outcomes.

## Data sharing statement

Further information on the data can be requested via email to the corresponding author.

**How does this paper make a difference in general practice?**

- With half of patients with diabetes intending to use diabetes self-management applications (DSMAs), there is a clear potential for these tools to become integral in diabetes care.
- Patients with higher incomes and those who are more open to change are more likely to adopt DSMAs. This insight can help practitioners tailor their approach, focusing on these demographics initially to build a strong user base and demonstrate the benefits of DSMAs to a broader audience.
- Emphasising the usefulness of DSMAs and addressing facilitating conditions can significantly increase patients’ intention to use these tools.
- This study underscores the importance of personalised patient education and targeted interventions in promoting the adoption of digital health tools.
